# Cotard's Syndrome in a Patient with Schizophrenia: Case Report and Review of the Literature

**DOI:** 10.1155/2016/6968409

**Published:** 2016-12-08

**Authors:** Jeff Huarcaya-Victoria, Mario Ledesma-Gastañadui, Maria Huete-Cordova

**Affiliations:** ^1^School of Medicine, Universidad Nacional Mayor de San Marcos, Lima, Peru; ^2^Department of Psychiatry, National Hospital Guillermo Almenara Irigoyen, Lima, Peru; ^3^School of Medicine, Universidad Nacional Federico Villarreal, Lima, Peru

## Abstract

Jules Cotard described, in 1880, the case of a patient characterized by delusions of negation, immortality, and guilt as well as melancholic anxiety among other clinical features. Later this constellation of symptoms was given the eponym Cotard's syndrome, going through a series of theoretical vicissitudes, considering itself currently as just the presence of nihilistic delusions. The presentation of the complete clinical features described by Cotard is a rare occurrence, especially in the context of schizophrenia. Here we present the case of a 50-year-old male patient with schizophrenia who developed Cotard's syndrome. The patient was treated with aripiprazole, showing improvement after two weeks of treatment. A review of the literature is performed about this case.

## 1. Introduction

On June 28, 1880, Jules Cotard gave a lecture at the* Société Médico-Psychologique* entitled* Du délire hypocondriaque dans une forme grave de la mélancolie anxieuse*, in which he described the case of a 43-year-old woman who claimed to have no brain, nerves, stomach, or soul and whose belief was that neither God or the devil existed, thus avoiding the need to eat and existing eternally until she was burnt [1, 2]. Cotard explained that the* délire hypocondriaque* resulted from a “…delusional interpretation of the pathological sensations experienced by patients suffering from common anxious melancholia” [2]. He classified this as a new subtype of* lypémanie*, whose original features Cotard described as (a) melancholic anxiety, (b) condemnation or possession ideas, (c) suicidal behavior and voluntary mutilation, (d) insensitivity to pain, (e) nonexistence ideas involving the whole body or any of its parts, and (f) delusions of immortality, being these the original clinical features Cotard described [3, 4]. Two years later, he wrote that the* délire hypocondriaque* happened when the* délire des negations* had been established: “I propose the name of delusions of negation to assign the condition of patients… in which the negative disposition has reached its highest level… have no head, nor stomach, some even have no body; if they are shown any object, a flower, a rose, they reply: it is not a flower, it is not a rose. In some of them, negation is universal, nothing exists anymore, they themselves are nothing,” “When the delusion of negation has been established, it aims at the patient's personality; the external world… adapts a hypochondriac form” [5].

In 1893 Emil Régis coined the eponym Cotard's syndrome, which was later popularized by Jules Séglas [4, 6]. Did Cotard try to describe a new disorder or rather a severe form of melancholia? Whatever the answer to this question may be, we can say that nowadays we refer to delusions of negation or nihilism as being synonymous with Cotard's syndrome, in which patients deny the existence of parts of their bodies or even their whole bodies, leading to denial of the world around them [7–9]. However, this current idea of delusions of negation or nihilism does not capture the original concept by Cotard, since it is not only about the belief of being dead but also about anxiety, agitation, severe depression, suicidal behavior and other delusive ideas. More so, Berrios [1] has concluded that the translation of* délire des negations* as nihilistic delusion has given the wrong interpretation that it is simply about the alteration of the thought process. Delgado [10] also realized the true dimension of Cotard's syndrome by classifying it as a rare form of chronic anxious melancholia whose characteristics encompassed systematic ideas of negation and negative enormity, hypochondriac convictions, ideas of nonexistence, immortality, and nonexistence of the world.

In order to briefly examine some characteristics of Cotard's syndrome, we present the case of a patient with schizophrenia who concurrently developed this syndrome. We describe the clinical course and response to treatment.

## 2. Case Report

It is about a 50-year-old male patient, native of Cajamarca, who had completed high school and lived with his son and daughter-in-law for three months. His family psychiatric background included a mother with bipolar affective disorder, a father who was a heavy drinker, and two sisters with depression diagnoses. None of them had presented Cotard's features in the course of their disorders.

Since the age of 13 the patient has presented behavioral changes, with marked isolation due to fearing other people and poor school performance so he repeated third year of high school. The patient completed his studies with difficulties and had no interest in continuing, so he decided to work on his parent's farm. His relatives described him as “strange,” “weird,” of “repressed character,” quiet, very dependent on his parents and as someone who took no initiative, ran no projects, did not bathe, and had no interest in personal hygiene. This behavior persisted and for periods got worse.

At age 45 the patient was afraid to leave home, as he thought he would be killed., He said that the police wanted to take him for all the bad things he had done, and he hid in the field, and did not want to stay alone. His relatives sent him to a hospital in Lima, where he was first diagnosed with schizophrenia. He was treated with olanzapine 5 mg/day, but the intake was irregular.

At age 49, the patient began to live alone in Cajamarca. He did not care about personal hygiene nor basic needs; he began giving away his belongings. He persisted with the ideas of police persecution. At some point he said that it would be better to surrender and confess all the bad things he had done and also requested a priest to confess his “great sins.” He could not sleep for several nights and was very anxious. At a hospital in Chiclayo, he was treated with olanzapine 10 mg/day and clonazepam 2 mg/day; with this therapy, he showed some improvement, was no longer anxious, and could go off home alone.

Three months before the admission to our unit, the patient moved to Lima with his son and started working in the cleaning area of a school. He had however a hard time adapting. He stopped taking olanzapine and clonazepam after fifteen days of treatment. He began to say that he was guilty for all the bad things he had done, as being unfaithful and attempting to rape his niece and sister-in-law. He felt scared and thought his relatives would report him and that he would go to jail.

Twenty days before admission to our unit, the patient refused to eat; he said the food was rotten as he was. Again, he was anxious, asked his relatives for help, and said there were people who wanted to kill him. He heard loud noises and began to see others' faces blurred, saying that it was because they were all dead and also said that he was dying because he was not in the “glory of Lord.” For all of these, he was taken to a psychiatric hospital in Lima, where he is treated with fluoxetine 10 mg/day and quetiapine 50 mg/day. He showed no improvement with this therapy.

Seven days before admission to our unit, the patient mentioned that he was already dead, his stomach did not work, his liver was decomposed, his brain was paralyzed, and his face lacked blood. He did not eat for fear of dying. The sounds got defined features, becoming imperative voices telling him “to leave this world, he was the devil.” Negativism emphasized, he did not eat, and his body weight diminished 4 kg. He stayed motionless in his bed most of the day and decreased his communication. He expressed his desire of dying to end all his suffering.

Four days before admission to our unit, the patient's relatives took him to a psychiatric hospital due to persistence of symptoms. He was again diagnosed with schizophrenia and admission was indicated.

The patient remained hospitalized for three days. During this time he received haloperidol 30 mg/day IM and clonazepam 3 mg/day. Since he did not show any improvement, his family decided to bring him to the emergency room of our hospital, before being admitted to the service of General Psychiatry.

On physical examination scaly lesions on the scalp were found, generalized rigidity, and short step gait. The rest of the somatic examination did not reveal any pathological findings. During mental examination, the patient was found in a pharaonic position, with narrow consciousness, confusion, paralysis of self, derealization, and depersonalization. He had an altered consciousness of existence and execution; his attention was easily fatigued; he had feelings of strangeness with goal frustration, imperative auditory hallucinations, visual illusions, and cenesthopathic delusions. He displayed precategorical thinking such as mental concretism, delusions of nonexistence, immortality, and condemnation. The speech was characterized by poverty and hypophonia. There was also flat affection, paranoid mood, ambivalence, and feelings of guilt. Finally, the patient displayed decreased vital energy, insomnia, hypokinesia, and waxy flexibility, maintaining persistent positions as well as lack of spontaneity.

In lab tests, alterations were found in vitamin B12:190 (reference values 200–900) pg/mL and Dimer-D: 1.45 (reference values <0.5) *μ*g/ml. Upper endoscopy showed esophagitis Los Angeles A. On brain computerized tomography (CT) scan no alterations were found.

The next diagnoses were proposed: (a) schizophrenia (295.90), (b) gastroesophageal reflux disease and esophagitis, and (c) seborrheic dermatitis. It was decided to start treatment with aripiprazole 30 mg/day and clonazepam 2 mg/day. The patient was significantly improved after two weeks of treatment. Delusions were diminished and emotional resonance improved.

## 3. Discussion

Berrios and Luque [12], when historically studying Cotard's syndrome in occidental psychiatric practice, analyzed 100 cases with this syndrome reported between 1880 and 1995. They found that 89% of these patients had depressive symptoms, 65% had anxiety, and 63% had feelings of guilt. Within nihilistic delusions, 86% concerned the body while those concerning their own existence were found in 69% of the cases. Hypochondriacal and immortality delusions were reported in 58% and 55% of the cases, respectively. Of the cases 22% reported auditory hallucinations and 19% visual hallucinations. When analyzing, three factors were found: (a) psychotic depression: anxiety, delusions of guilt, depression, and auditory hallucinations; (b) Cotard type I: hypochondriacal delusions, nihilistic delusions concerning the body, concepts, and existence; and (c) Cotard type II: anxiety, delusions of immortality, auditory hallucinations, nihilistic delusions concerning existence, and suicidal behavior. According to Berrios and Luque [12], type I will establish the pure form of Cotard's syndrome, being delusional in origin and not secondary to the affective disorder. All of this would have therapeutic implications because these patients would not respond to antidepressant treatment.

This syndrome is considered to be rare. Ramirez-Bermudez et al. [13] reported that out of 479 Mexican patients with a primary psychiatric disorder, including 150 patients with schizophrenia, three had Cotard's syndrome (0.62%) and psychotic depression. In 2013, Stompe and Schanda [15] reviewed 346 cases of schizophrenia and found three patients with Cotard's syndrome (0,87%). If the occurrence of Cotard's syndrome in the context of depression is unusual, it is even more unusual among patients with schizophrenia spectrum disorders. The association between Cotard's syndrome and schizophrenia may increase the risk of self-aggressive behavior [16, 20–22]. There are also reports of cases with this syndrome in other mental disorders and neurological diseases (Table 1).

According to Coltheart et al. [40], Cotard's syndrome is seen as a monothematic delusion, a concept we consider erroneous. If we want to have a clear idea of the clinical presentation of this syndrome, we must review the clinical descriptions given by Cotard [5] and Séglas [6], among others. Consider our patient's symptoms:

(a) Delusions of negation are the most representative symptom of Cotard's syndrome. Negation may affect the somatic constitution of the patient; the metaphysical representations were created on their own as what has happened with our patient. It differs from persecutory delusions, where patients are great ontologists, creating rich worlds and new metaphysical identities [1]. In other cases, negation projects to the exterior world, which is also seen in our patient when reporting people around him as dead. Altering the consciousness of existence as loss of sense of self is referred to as an alteration of the consciousness of common self in Cotard's syndrome. For Saavedra [41] this can also occur in schizophrenia, being disfigured by hallucinatory phenomena and delusions of it. Saavedra [41] proposed the name “Pseudo-Cotard's syndrome” to this presentation, considered as a variant form of cenesthopathic schizophrenia. These patients break with the philosophy of Descartes [32], for whom one can doubt everything, less of its own existence: “Je pense, donc je suis.”

(b) Delusions of immortality: we noticed that our patient mentioned he was dead but was afraid of dying. This situation, apparently paradoxical, was described as a form of delusion of immortality in the original works of Cotard [5] and Séglas [6]. When hospitalized, our patient mentioned that he indeed was immortal. These delusions of immortality may constitute forms of megalomaniac hypochondriac delusions [2, 6].

(c) Delusions of condemnation were shown as constant self-accusations in our patient. Everything that happened was a form of divine punishment to atone his “great sins.” Of interest is also the content of verbal hallucinations, according to which our patient was the devil himself. For Séglas [6], the delusions of possession happen when the patient considers herself/himself as a demon (demonomania) or when she/he is subjected to an internal power and cannot direct her/his own actions (internal demonopathy).

(d) Disturbed sensation is considered as one of the most important phenomena in psychopathology due to its role in the development of delusions. Analgesia, hyperalgesia, and paralgesia are found. In addition, disturbances in visceral sensation may be present; for instance, our patient reported generalized malaise related to anatomical and functional conditions of his internal organs (“his liver was rotten”), the delusive perception of a persistent gastric problem, which he said led to his death, and had its origin in gastroesophageal reflux disease. With regard to the circulatory and nervous systems (“he lacks blood in his face,” “his brain was paralyzed”), we did not find any disturbance that could explain these delusive perceptions. In addition, there are several situations concerning organ perception; for example, Séglas [6] reported abnormal sensations in the groins that can be observed in some cases of Cotard's syndrome and Koro [42, 43].

(e) Many of the following phenomena were found in our patient: mutism with modifications of language, food refusal, suicidal ideation and suicide, or voluntary mutilation [6].

**Figure 1:**
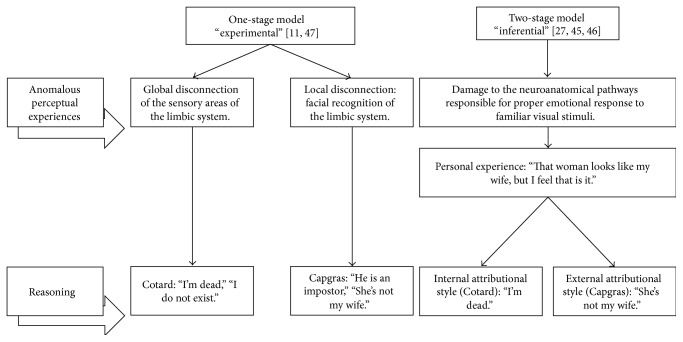
Theoretical explanations of Cotard and Capgras delusions. Elaborated from [11].

The theoretical explanations to the delusions in this syndrome vary (Figure 1) and can be divided into two groups: (a) a “one-stage” or experimental model, where delusions may be normal reasoning in response to an abnormal perception and (b) a “two-stage” or inferential model, where delusions are caused by abnormal reasoning in response to an abnormal perception [44]. Young et al. [27, 45] defend the two-stage model. They believe that delusions in Cotard's and Capgras syndromes are related due to their neurological and psychological similarities in reported cases. They propose that delusions in Cotard's and Capgras syndromes reflect an interaction of failures at two levels: abnormal perceptual experiences and an incorrect interpretation of these. The authors suggest that delusions in Capgras syndrome are due to damage to the neuroanatomical pathways responsible for proper emotional response to familiar visual stimuli. A similar process could be responsible for the delusions of Cotard's syndrome, in which patients say that they “feel nothing inside,” so the essential difference between these delusions would not be the experience, but the wrong forms in which they are rationalized, being in Capgras an external attributional style (“He is an impostor”), while in Cotard an internal attributional style (“I'm dead”) [46]. On the contrary, Gerrans [11] defends the one-stage model; he argues that delusions can only be explained in terms of an abnormal experience and that the apparently abnormal reasoning shown in the two-stage model describes normal reasoning processes. He also criticizes the proposal of Young et al. (that Cotard's and Capgras delusions would be intimately related). Gerrans [11] cites Ramachandran and Blakeslee [47], for whom both these delusional experiences are fundamentally different due to their lack of affective response in several areas. In Cotard's syndrome the absence of a global affective response would happen due to a disconnection of all sensitive areas of the limbic system, resulting in a complete lack of emotional contact with the world. In Capgras syndrome however this would be restricted to facial recognition.

Apart from the delusional ideas we found catatonic symptoms in our patient. This combination is very rare and has been reported in only a few cases [24–26]. It is important to emphasize the elevation of Dimer-D (1.45 *μ*g/ml) without any organic cause. As already reported on a catatonic syndrome [14], some authors conclude that in the absence of organic cause and in presence of motor, affective and behavioral disturbances that mimic catatonic features and high levels of Dimer-D (>0.5 *μ*g/ml) may be suggestive of a catatonic diagnosis.

Treatment for Cotard's syndrome focuses on its clinical origin. Thus, antidepressants can be useful in patients with affective disorders [48], and there are studies which show that electroconvulsive therapy (ECT) was successful [29, 49–51], leading to proposing it as the treatment of choice. Antipsychotic drug treatment or combination strategies (antipsychotics plus antidepressants) are also used. In a case report, Chou et al. [52] observed a dramatic improvement of symptoms in a patient with Cotard's syndrome after two months of treatment with fluoxetine 40 mg/day and risperidone 6 mg/day. Another reported combination [53] was venlafaxine 225 mg/day and quetiapine 600 mg/day, which produced relief of depressive symptoms and delusions of negation in a 68-year-old patient after two weeks of treatment. In yet another case report [54] clozapine 50 mg/day, fluvoxamine 200 mg/day, and imipramine 50 mg/day led to a complete remission of delusions in five days.

Monotherapy treatment with sulpiride 300 mg/day was reported as successful in a 33-year-old patient with schizophrenia who developed the Cotard's syndrome, and Shiraishi et al. [55] concluded that treatment with sulpiride is the first line for Cotard's syndrome in schizophrenia. If we follow the classification system proposed by Berrios and Luque [12], our patient follows the criteria of Cotard type I, since no depressive symptoms became evident. Thus, the syndrome was considered delusional in origin and not secondary to an affective disorder and we decided to start with aripiprazole up to 30 mg/day. During this treatment we observed an improvement of psychotic symptoms, with a decreased intensity of delusions after two weeks (the patient said that he was no longer dead). The patients also improved with regard to social contact and negative symptoms. Being improved the patient was discharged after four weeks of hospitalization. There are reports in which aripiprazole, both in monotherapy or combined with other psychopharmacological drugs, showed effectiveness in Cotard's syndrome [56–59]. De Risio et al. [54] kept a record of hyperactivity in dopaminergic systems related to psychosis in a patient with Cotard's syndrome. This would explain the effectiveness of antipsychotic treatment in some patients with Cotard's syndrome, especially type I. Aripiprazole's effectiveness relies on its mechanism of action: partial agonist at dopamine D2, dopamine D3, and serotonin 1A receptors and antagonist at serotonin 2A receptors. All of these produce stabilization of the dopaminergic and serotoninergic systems, which explains its antidepressant and antipsychotic effect.

Regarding the psychotherapeutic treatment of Cotard's syndrome, there are few reported cases since most reports focus on pharmacotherapy. Bott et al. [22] reported the case of a patient with schizophrenia who developed Cotard's syndrome. According to the authors, this case provides anecdotal evidence of the effectiveness of combining pharmacotherapy with cognitive behavior therapy.

## 4. Conclusion

Cotard's syndrome is a rarity in psychiatry. The psychopathology exceeds by far the unique association with the nihilistic delusions that have been emphasized the last years; we can also find delusions of immortality, enormity, guilt, possession, and persecution, with psychopathological alterations of affection and will. Regarding treatment, the combination of antipsychotics and antidepressants is often used, but if this shows no improvement, ECT is suggested.

## Figures and Tables

**Table 1:** Mental disorders and neurological diseases observed in patients with Cotard's syndrome.

*Mental disorders*
Depressive disorder [12–14]
Schizophrenic spectrum [15–22]
Bipolar disorder [23]
Catatonia [24–26]
Capgras syndrome [18, 27, 28]
Lycanthropy [23, 29]
*Neurological diseases*
Ischemic cerebrovascular disease [28, 30–32]
Subdural hemorrhage [33]
Parkinson's disease [34]
Traumatic brain injury [35]
Multiple sclerosis [36]
Arteriovenous malformation [36]
Epilepsy [37]
Semantic dementia [38]
Atrophy of the insular cortex [39]
